# Assessment of the Probability of Post-thrombotic Syndrome in Patients with Lower Extremity Deep Venous Thrombosis

**DOI:** 10.1038/s41598-018-30645-w

**Published:** 2018-08-23

**Authors:** Hao Huang, Jian-Ping Gu, Hao-Fan Shi, Wan-Yin Shi, Jing-Yuan Lu, Liang Chen, Hao-Bo Su

**Affiliations:** 1 Department of Interventional Radiology, Nanjing First Hospital, Nanjing Medical University, Nanjing, 210001 China; 2 Obstetrics and Gynecology Hospital Affiliated to Nanjing Medical University, Nanjing, 210004 China

## Abstract

This study was performed to assess the probability of post-thrombotic syndrome (PTS) after treatment of lower extremity deep venous thrombosis (LEDVT). Patients with LEDVT undergoing their first treatments in Nanjing First Hospital from January 2013 to December 2014 were enrolled in this study (156 patients were enrolled in the training cohort, and 135 patients were enrolled in the validation cohort). 51 and 45 patients developed PTS in the two cohorts, respectively. Independent risk factors for PTS were investigated in the training cohort, and these independent risk factors were employed to develop the APTSD scoring system with which to predict the probability of PTS. Four independent risk factors for PTS were identified: iliac vein compression syndrome, residual iliac-femoral vein thrombosis, residual femoral-popliteal vein thrombosis and insufficient anticoagulation. Patients in the training cohort were divided into 2 groups according to the APTSD score of ≤7.0 and >7.0 points regarding the probability of PTS (median PTS-free time, 21.82 vs. 18.84 months; P < 0.001). The accuracy of this score system was 81.7% for the training cohort and 82.5% for the validation cohort. Patients with an APTSD score of >7.0 points may have an increased probability of developing PTS.

## Introduction

Post-thrombotic syndrome (PTS) is the most common complication in patients with lower extremity deep venous thrombosis (LEDVT), which can be seen in 20% to 50% of patients with acute LEDVT^1–3^. Symptoms of PTS usually occur between 1to 24 months after LEDVT^1^. Typical symptoms include cramping, pain, swelling, and heaviness in the leg and are aggravated when standing or walking. PTS causes pain and sufferings in affected patients and incurs a large burden on society^4^. Many studies^2,5,6^ have addressed the risk factors for PTS after LEDVT, such as age, body mass index(BMI), residual obstruction and insufficient anticoagulation treatment. However, no study has provided a scoring system to assess the probability of PTS. Based on a priori hypotheses or published reports^5,7–9^, interventional physicians used a standardised form, to record demographic and clinical characteristics considered to be plausible predictors of PTS. The data included (1) demographics, such as sex, age and body mass index(BMI); (2) clinical characteristics, such as current smoking, hypertension, diabetes and chronic heart disease; (3) characteristics of index LEDVT, such as time since index LEDVT diagnosis, D-dimer, affected leg, proximal LEDVT, iliac vein compression syndrome, symptomatic pulmonary embolism and provoking features of LEDVT (cancer-related, trauma, surgery, or immobilization ≥3 days in the past 3 months); and (4) parameters related to the treatment, such as filter implantation, residual vein thrombosis, insufficient anticoagulation and compression stockings use. We therefore conducted a retrospective single-centre study to assess the probability of PTS on patients with a first time LEDVT and help fill these knowledge gaps.

## Methods

This study was approved by the Ethical Review Committee of Nanjing First Hospital, Nanjing Medical University. The methods were carried out in accordance with the approved guidelines. Blood was collected and procedures were carried out in accordance to the relevant guidelines of the ethics committee. We adhered strictly to the Declaration of Helsinki.

### Patient Inclusion and Exclusion Criteria

Patients who met the following inclusion criteria^10^ were enrolled in this study: (1) patients aged over 18 years^2,11^, (2) first-ever diagnosis of LEDVT,(3) symptoms that had lasted <28 days^12^, and (4) provision of informed consent. Patients who met the following exclusion criteria^10,12,13^ were excluded from the study: (1) previous diagnosis of LEDVT, (2) severe renal failure with an estimated creatinine clearance rate of <30 mL/min, (3) life expectancy of no >24 months, (4) drug abuse or mental disease that might interfere with treatment and follow-up, and (5) incomplete medical records or follow-up data.

### Definition of Clinical Outcome and Data Assessment

Diagnosis of LEDVT was verified by compression ultrasound. In some inconclusive cases, the diagnosis was confirmed through supplementary venography or computed tomography venography. Computed tomography venography was necessary for patients with iliac vein compression syndrome (IVCS). Iliac vein compression was qualitatively estimated by asking the radiologists to rate the severity of common IVCS^7^. Venous imaging data from patients, including the occurrence of LEDVT, were retrospectively reviewed by two interventional physicians (L.C. and W.Y.S.) using a PACS system (FIRSTECH PACS; Anhui Firstech Co., Ltd., Hefei, China). Both interventional physicians had more than 5 years of experience in diagnostic radiology. Any disagreement between the two interventional physicians was resolved by consensus with another physician (J.P.G.). Patients with proximal LEDVT were more likely to benefit from Catheter-directed thrombolysis (CDT) and those want to prevent PTS are more likely to choose CDT over anticoagulation only. All therapeutic protocols, including CDT, anticoagulation, angioplasty and insertion of stents, were decided by both interventional physicians. Anticoagulation was routinely initiated in all patients on the day of diagnosis according to international guidelines^8^. Low-molecular-weight heparin (subcutaneous injection of either nadroparin at a dose of 85 IU/kg [Fraxiparine; GlaxoSmithKline, Brentford, UK] or enoxaparin at a dose of 1.5 mg/kg [Sanofi, Paris, France]) was given in combination with oral warfarin for at least 5 days, until the target international normalised ratio (INR) of 2.0 to 3.0 was reached. All patients were given a pair of knee-high elastic compression stockings (class II) and were advised to wear a stocking on the index leg daily for at least 2 years. We also advised replacing the pair of stockings every 6 months.

All patients with LEDVT in our department had paid regular follow-up visits 1 month (±1 week), 3 months (±2 weeks), 6 months (±2 weeks), 12 months (±3 weeks) and 2 years (±4 weeks) after treatment. During the follow-up visits, the patients were interviewed and examined by a vascular physiologist with no previous knowledge of the patients’ treatment allocation. Insufficient anticoagulation was defined as anticoagulation that was subtherapeutic^9^ for >20% of the time. PTS was assessed using the Villalta scale, as recommended by the International Society on Thrombosis and Haemostasis^14^. All patients in our department were told to contact the vascular physiologist if their symptoms bacame markedly aggravated; e.g., if they developed pain (spontaneous or during ambulation), cramps, pruritus or paresthesia. In such cases, the vascular physiologist immediately assessed the patients using the Villalta scale^15^. The Villalta scale is used to assess five patient-rated venous symptoms and six clinician-rated physical signs. A Villalta score of >5 points or a lower limb venous ulcer is classified as PTS^15^. Patients who developed new symptoms between the follow-up periods and could not determine the time point at which the symptoms had occurred were excluded from this study.

### Statistical Analysis

PTS probability curves were calculated using the Kaplan-Meier method. Factors with a P-value of <0.200 in univariate analysis were adjusted for as potential confounding factors. The variables in the final model were subsequently incorporated into a stepwise Cox regression model as candidate variables. The regression coefficients of the Cox regression model were multiplied by 5 and rounded to facilitate, the use of point numbers and thus calculate the APTSD score. All statistical analyses were performed using PASW Statistics software (Version 18.0, IBM Corporation, Armonk, NY, USA).

## Results

### Patient Characteristics

**Figure 1:**
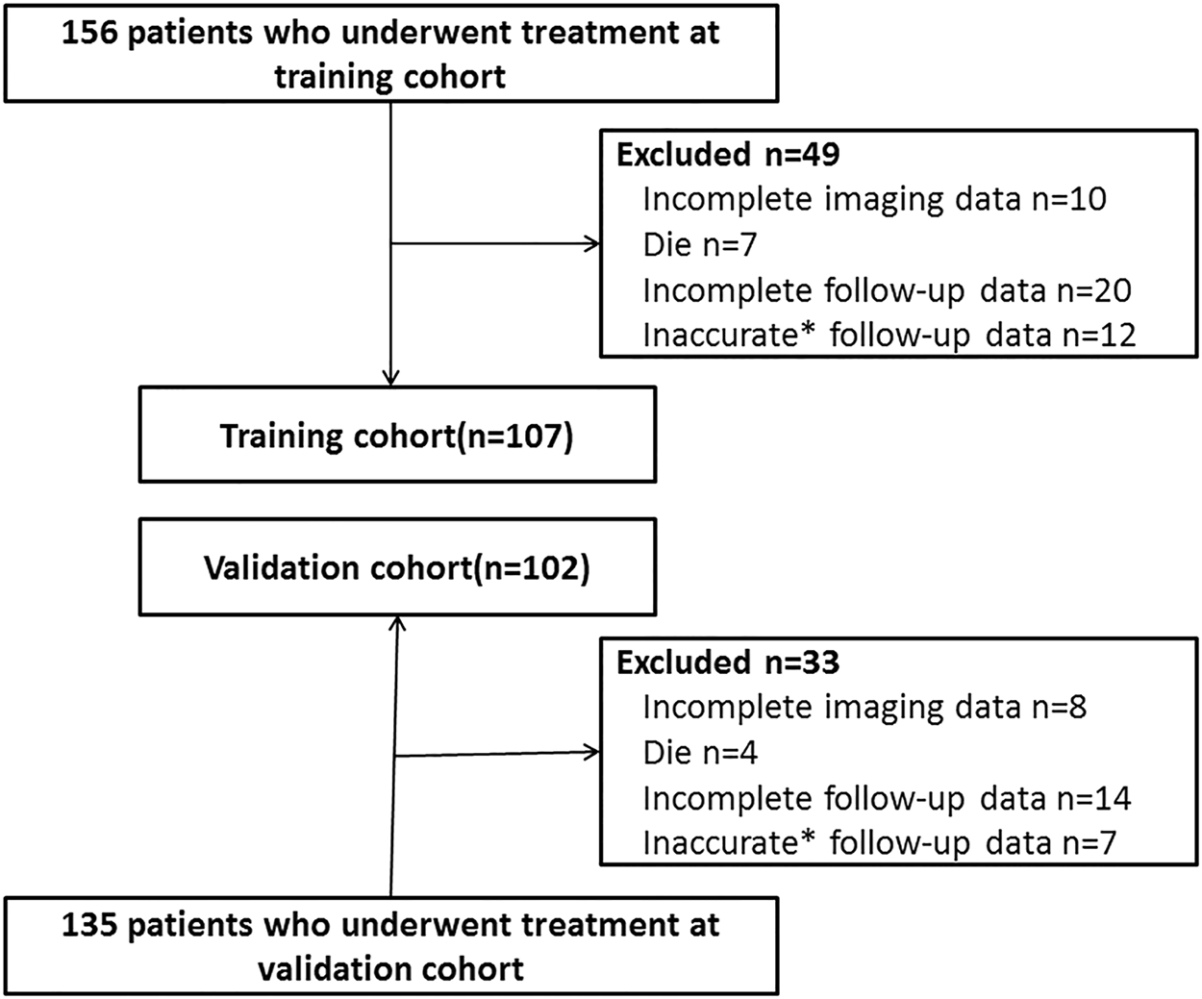
Patient selection flow chart. *Patients who developed new symptoms between the follow-up periods and could not determine the time point at which the symptoms had occurred were excluded from this study.

In total, 156 patients who underwent treatment at Nanjing First Hospital (training cohort) from January 2013 to December 2013 were screened to establish the objective risk scoring system. Additionally, 135 patients who underwent treatment at Nanjing First Hospital (validation cohort) from January 2014 to December 2014 were selected to assess the objective risk scoring system (Fig. 1). A total of 146 patients in the training cohort met the inclusion criteria. Of these, 7 died during follow-up (unrelated to LEDVT), and another 32 were excluded because of incomplete (20 patients) or inaccurate (12 patients) follow-up data. Therefore, a total of 107 patients in the training cohort were analyzed. A total of 127 patients in the validation cohort met the inclusion criteria. Of these, 4 died during follow-up (unrelated to LEDVT), and another 21 were excluded because of incomplete (14 patients) or inaccurate (7 patients) follow-up data. Consequently, 102 patients in the validation cohort were included in the analyses. The demographic and clinical characteristics of both cohorts are presented in Table 1. The two groups showed a significant difference in the category “Index LEDVT+ Pulmonary Embolism (vs. LEDVT alone)” (P < 0.05).

**Table 1:** Patient Characteristics.

Characteristic	Training Cohort (n = 107)	Validation Cohort (n = 102)	P-value
Gender, n (%)			0.962
Male	50(46.7%)	48(47.1%)	
Female	57(53.3%)	54(52.9%)	
Acute (vs. Subacute) stage, *n (%)*	55(51.4%)	56(54.9%)	0.612
Age, *years*	54.40(17.67)	55.05(18.93)	0.224
Affected leg, *n (%)*			0.849
Left	70(65.4%)	68(66.7%)	
Right	37(34.6%)	34(33.3%)	
Proximal LEDVT, *n (%)*	88(82.2%)	81(79.4%)	0.603
Iliac compression syndrome, n *(%)*	50(46.7%)	52(51.0%)	0.539
Index LEDVT + PE (vs. LEDVT alone), *n (%)*	15(14.0%)	27(26.5%)	0.025
Current smoker, *n (%)*	21(19.6%)	22(21.6%)	0.728
Body mass index, *kg/m*^2^	24.16(3.08)	23.78(3.55)	0.070
Patients with PTS, *n(%)*	51(47.7%)	45(44.1%)	0.607

Chi-square test or one-way ANOVA was used. Data are mean (SD) or number (%). PE = Pulmonary Embolism.

In total, 107 patients in the training cohort were diagnosed with their first-ever LEDVT; of these patients, 51 (47.7%) developed PTS. Forty-five of the 102 patients with LEDVT in the validation cohort developed PTS (44.1%).

### **Univariate Analysis of Risk Factors** in Training Cohort

The median PTS-free time in the training cohort was 20.57 (standard deviation, 0.61) months. As a result, seven risk factors with P-value of <0.200 were adjusted for as potential confounding factors: acute stage, proximal LEDVT, IVCS, residual iliac-femoral vein thrombosis, residual femoral-popliteal vein thrombosis, filter implantation, and insufficient anticoagulation(Table 2).

**Table 2:** Univariate analysis of risk factors for the newly developed PTS.

Characteristic	HR	95%CI	P-value
Gender	0.917	0.527–1.596	0.759
BMI	1.035	0.941–1.139	0.475
Acute	0.640	0.368–1.112	0.113
Proximal LEDVT	2.468	0.980–6.215	0.055
Provoking features of LEDVT^a^	1.135	0.639–2.016	0.665
IVCS^b^			
No	1		
Severe	1.451	0.633–3.323	0.379
Occlusion	3.010	1.626–5.574	<0.001
Residual Iliac-Femoral vein thrombosis^b,c^	2.258	1.408–3.621	0.001
Residual Femoral-Popliteal vein thrombosis^b,d^	2.755	1.537–4.937	0.001
D-dimer	0.994	0.967–1.021	0.657
Filter implantation			
No	1		
Temporary	1.055	0.548–2.029	0.873
Permanent	1.749	0.807–3.790	0.157
Current smoker	1.089	0.570–2.080	0.797
Insufficient Anticoagulation	2.230	1.268–3.923	0.005
Compression stockings use	1.063	0.613–1.842	0.828

BMI: Body Mass Index.

IVCS: Iliac Vein Compression Syndrome.

^a^Provoking features of LEDVT were defined as surgery, trauma, cancer, or immobilization for 3 or more days during the past 3 months.

^b^Diagnosis for patient whose therapeutic schedule included catheter-directed thrombolysis, angioplasty or insertion of stents was confirmed based on the last venography or CT venography after these treatments.

^c^Residual iliac-femoral vein thrombosis was defined as residual iliac vein and/or common femoral thrombosis at the patients’ last venography before discharged.

^d^Residual femoral-Popliteal vein thrombosis was defined as residual femoral and/or popliteal vein thrombosis at the patients’ last venography before discharged.

### Verification of Independent Risk Factors for PTS in Training Cohort and Establishment of APTSD Scoring System

The above-mentioned seven factors with a P-value of <0.200 were assessed by Cox regression analysis to eliminate any potential confounding factors. Of these seven factors, the following four risk factors were chosen as the candidate predictors: iliac vein compression (iliac vein occlusion), residual iliac-femoral venous thrombosis, residual femoral-popliteal vein thrombosis and non-standard anticoagulation. These four variables were chosen because they had the greatest effect on the result. Finally, these four variables were verified by Cox regression analysis as independent factors predicting PTS (Table 3). Moreover, the regression coefficients (B-values) in the Cox regression model were multiplied by 5 and rounded to facilitate the use of point numbers and thus calculate the APTSD score (Table 3).

**Table 3:** Multivariate backward stepwise Cox regression analysis of risk factors for the newly developed PTS in patients in training cohort.

Variable	HR	95%CI	B	APTSD scores	P-value
IVCS					
No	1		0	0	
Severe	1.612	0.696–3.733	0.478	2.5	0.265
Occlusion	2.983	1.534–5.801	1.093	5.5	<0.001
Residual Iliac-femoral vein thrombosis					
No	1		0	0	
Yes	1.929	1.008–3.422	0.657	3	0.025
Residual Femoral-Popliteal vein thrombosis					
No	1		0	0	
Yes	1.881	0.987–3.586	0.632	3	0.055
Insufficient Anticoagulation					
No	1		0	0	
Yes	3.192	1.725–5.905	1.161	6	<0.001

IVCS: Iliac Vein Compression Syndrome.

**Figure 2:**
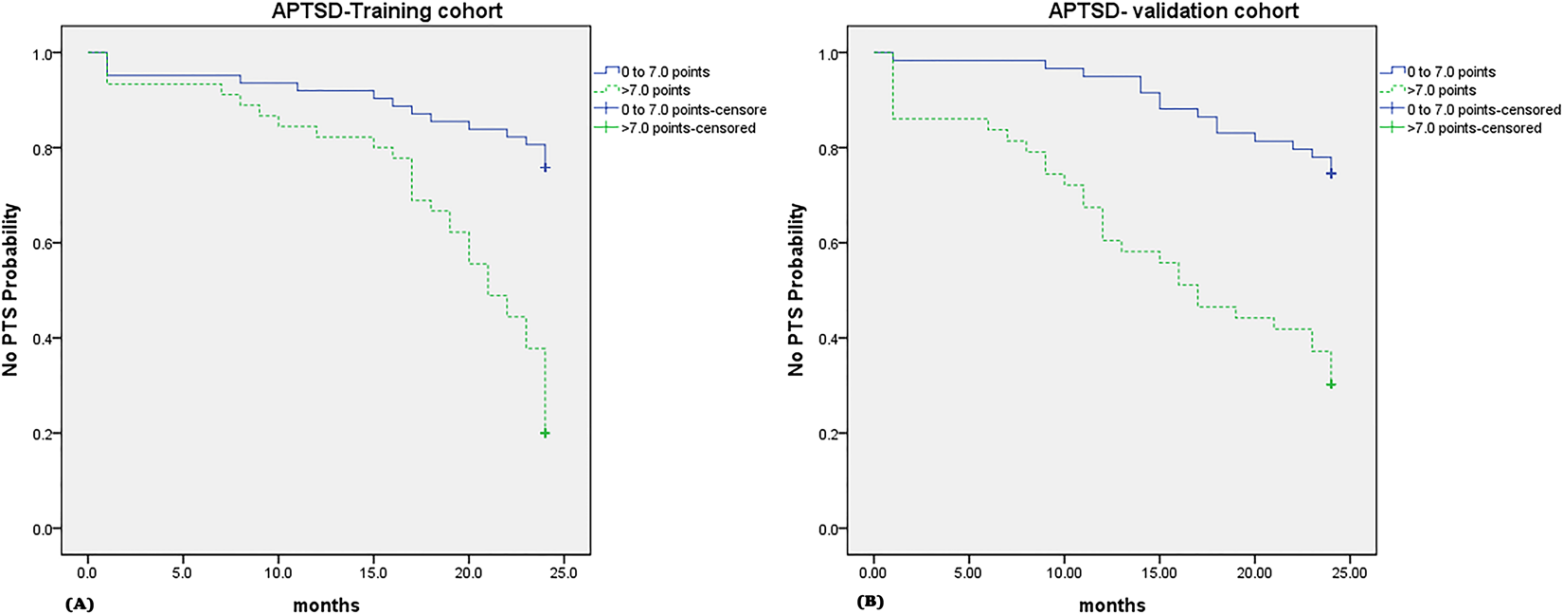
(**A**,**B**) (**A**) No PTS probability of both APTSD score groups (0 to 7.0 points and >7.0 points) in training cohort. (**B**) No PTS probability of both APTSD score groups (0 to 7.0 points and >7.0 points) in validation cohort. All analyses were performed using the Kaplan-Meier method and Log-rank test.

The APTSD scores of patients in the training cohort (n = 107) were calculated using the four independent risk factors. The chi-square value obtained by the Hosmer–Lemeshow test was 39.089 (P < 0.001). Receiver operating characteristic curves(ROC) were used to determine the best cut-off point in terms of maximum of sensitivity and specificity (Supplement 2). The largest significant difference was observed between patients with an APTSD score of <7.0 points (n = 62) and >7.0 points (n = 45) (21.82; 95% confidence interval [CI], 20.37–23.28 months) vs. 18.84; 95% CI, 16.86–20.83) months, respectively; P < 0.001] (Fig. 2A). Patients with an APTSD score of <7.0 points exhibited a higher 1-year PTS-free probability than those with a score of >7.0 points (81% vs. 38%, respectively; P < 0.001). Furthermore, the 2-year fracture-free probabilities for those two groups were 72% and 14%, respectively (P < 0.001). A total of 75.8% of patients with an APTSD score <7.0 points developed no PTS during the follow-up period, while 20.0% of patients with an APTSD score of >7.0 points developed no PTS. As stated above, the patients were divided into 2 groups based on an APTSD score of <7.0 points and >7.0 points. Furthermore, correct predictions were made for patients without PTS in both the low-score group (<7.0 points) and high-score group (>7.0 points) (Table 4). We calculated the “model accuracy” to assess the accuracy of the APTSD score system. The definition of “model accuracy” was the percentage of patients for whom the correct prediction was made among the total patients. Thus, the general accuracy in the training cohort was 81.7% (Supplement 2).

**Table 4:** Post-thrombotic syndrome in two groups.

	Training Cohort			Validation Cohort		
	Low-score Group (n = 62)	High-score Group (n = 45)	P-value	Low-score Group (n = 59)	High-score Group (n = 43)	P-value
PTS	15(24.2%)	36(80.0%)	P < 0.001	15(25.4%)	30(69.8%)	P < 0.001
PTS Severity Category						
Mild (score 5–9)	11(17.7%)	28(62.2%)		13(22.0%)	24(55.8%)	
Moderate (score 10–14)	4(6.5%)	5(11.1%)		2(3.4%)	3(7.0%)	
Severe (score >14 or lower limb venous ulcer)	0(0%)	3(6.7%)		0(0%)	3(7.0%)	

Chi-square test was used. Data are n(%).

PTS: Post-thrombotic syndrome.

Next, the APTSD scores of patients in the validation cohort were calculated (Table 4). The median PTS-free time was 22.05 months (95% CI, 20.86–23.24 months) in patients with an APTSD score of <7.0 points (n = 59) and 15.74 months (95% CI, 13.21–18.28 months) in those with a score of >7.0 points (n = 43) (P < 0.001) (Fig. 2B). The 1-year fracture-free probabilities in the above two groups were 78% and 37%, respectively (P < 0.001). Furthermore, the 2-year fracture-free probabilities in the two groups were 71% and 25%, respectively (P < 0.001). A total of 74.6% of patients with an APTSD score of <7.0 points did not develop PTS during the follow-up period, and 30.2% of those with an APTSD score of >7.0 points did not develop PTS. The general accuracy in the validation cohort was 82.5% (Supplement 2).

## Discussion

Approximately 20% to 50% of patients with LEDVT^1,2^ will develop PTS within 2 years of diagnosis; however, the exact risk factors for PTS remain uncertain. PTS was found in 45.9% of the patient population in the current study. The potential risk factors reported in the literature, such as age, BMI, thrombus location, residual obstruction and insufficient anticoagulation treatment, are considered to be strongly or moderately correlated with PTS. All of these risk factors were considered in the present analysis. However, the CDT and pharmacomechanical CDT(PCDT) were not considered. Anticoagulation is a basic treatment administered to every patient with LEVDT and is continued for several months, but other treatments are only used in inpatients. CDT or PCDT is used to reduce residual thrombosis. Angioplasty and stent implantation are used to treat IVCS. The details of these treatments (e.g., thrombolytic dose, stent size) differ among doctors, and the treatment results can be obtained before the patient is discharged from the hospital. Therefore, we analysed postoperative risk factors (residual thrombosis and IVCS) instead of treatment-related risk factors (CDT, PCDT and angioplasty). However, the correlation of other risk factors with PTS^16^, such as thrombophilia and sex, remain a source of debate. In the present study, iliac vein compression, residual iliac-femoral vein thrombosis, residual femoral-popliteal vein thrombosis and insufficient anticoagulation were found to be the independent risk factors for PTS.

Sub-acute (vs. acute) stage and proximal LEDVT are indicated in some studies to be one of the risk factors for PTS. Siddiqui *et al*.^5^ and Kahn *et al*.^1^ suggested that proximal LEDVT was remarkably correlated with PTS and they are potential confounding factors in our univariate analysis, however, only anticoagulation treatment was employed in their studies. Patients with acute proximal LEDVT receiving CDT or PCDT could have a lower risk of PTS, as shown in the CaVenT trial^17,18^. Thus, sub-acute and proximal LEDVT were excluded in the following analysis.Conversely, residual obstruction has been found to be strongly correlated with PTS in many studies^19,20^. Residual obstruction in a lower extremity vein may develop for many reasons, the most common of which are iliac vein compression and residual thrombosis. Both iliac vein compression and residual thrombosis were independent risk factors for PTS in the present study. CDT can produce good results in patients with a first episode of acute iliofemoral vein thrombosis. However, CDT cannot resolve a residual venous outflow obstruction or stenosis. Additionally, one study^21^, showed that stenting of an iliac vein obstruction following CDT in patients with LEDVT and IVCS may increase the patency of the deep vein, which may subsequently prevent the incidence of PTS.

In the current study, oral warfarin was administered based on the AT9 recommendations^8^. As shown in two studies, a subtherapeutic INR during the first 3 months of therapy will increase the risk of PTS. The first of these two studies^9^ reported an odds ratio of 1.84 for PTS in patients whose INR was subtherapeutic >20% of the time, whereas the second study^22^ showed that patients whose INR was subtherapeutic >50% of the time had a 2.7-fold higher risk of PTS. In the current study, patients whose INR was subtherapeutic >20% of the time had a 2.23-fold higher risk of PTS. Non-vitamin K oral anticoagulant (NOAC) is recommended by AT10^13^ as long-term anticoagulation instead of vitamin K antagonist (VKA) therapy in patients with LEDVT or pulmonary embolism but no cancer. NOAC may reduce the incidence of PTS; however, it should be verified in more studies in the future.

An association of PTS with the BMI, has been supported by some studies^1,20^, and the BMI is an established risk factor for PTS. In addition, some studies have proven that^5,23^, patients with a BMI of >35 kg/m^2^ are linked have a 6-fold higher probability of developing PTS. However, another study^24^ showed that the mean or median BMI is lower than non-Asian populations. In the current study, no patient had a the BMI of >35 kg/m^2^. Therefore, whether BMI is a risk factor for PTS in Asian populations should be further verified in further studies.

To the best of our knowledge, this is the first study attempting to predict the probability of PTS in patients with their first diagnosis of acute or subacute LEDVT by establishing a scoring system. Such a scoring system was established based on the regression coefficients of significant variables from a Cox regression model. Several disease models^25–27^ have demonstrated that, establishing a scoring system is a reasonable and feasible approach. The APTSD score has identified two distinct groups of patients with respect to the PTS-free time. Importantly, this scoring system, which was established based on patient characteristics in the training cohort, was applied in an independent external validation cohort. Moreover, the four independent risk factors identified in the current study can also be easily measured in daily practice.

This study has two main limitations. First, this was a retrospective, single-centre study. A prospective cohort in another setting is needed to validate the accuracy of this scoring system. Second, in patients with LEDVT or pulmonary embolism and no cancer, AT10^13^ suggests the use of NOACs over vitamin K antagonists for long-term anticoagulant therapy. More patients with NOACs should be considered in the future studies.

## Conclusion

In summary, the current study has verified that iliac vein compression, residual iliac-femoral vein thrombosis, residual femoral-popliteal vein thrombosis, and insufficient anticoagulation are independent risk factors for PTS. Moreover, patients with their first diagnosis of acute or subacute LEDVT with an APTSD score of >7.0 points may have an increased probability of developing PTS. The accuracy of this scoring system was 81.7% and 82.5% in our training cohort and validation cohort, respectively.

## Electronic supplementary material

Supplement-1

Supplement-2
