# Supplementary material for: Assessment of the Probability of Post-thrombotic Syndrome in Patients with Lower Extremity Deep Venous Thrombosis
Source: Sci Rep. 2018 Aug 23;8:12663. doi: 10.1038/s41598-018-30645-w (PMC6107662; doi:10.1038/s41598-018-30645-w)
Supplement: Supplementary file 1 — Supplement-1 [file 41598_2018_30645_MOESM1_ESM.pdf]

Assessment of the Probability of Post-thrombotic Syndrome in Patients with Lower Extremity Deep Venous Thrombosis

Hao Huang, MD[1],Jian-Ping Gu,MD[1], Hao-Fan Shi,MD[1],Wan-Yin Shi, MD[1], Jing-Yuan Lu, MD[2], Liang Chen, MD[1], Hao-Bo Su, MD[1]

[1]Department of Interventional Radiology, Nanjing First Hospital, Nanjing Medical University, Nanjing, 210001,China

[2]Obstetrics and Gynecology Hospital Affiliated to Nanjing Medical University, Nanjing, 210004, China

Address for Correspondence: Hao-Bo Su, MD, Department of Interventional Radiology, Nanjing First Hospital, Nanjing Medical University, 68 Changle Road , Nanjing, 210001,China

E-mail:nj_dsaalex@126.com

Supplement 1 Patient Characteristics

Characteristic	With PTS (n =96)	Without PTS (n =113)	P-value
Gender , n (%)			0.264
Male	41(42.7%)	57(50.4%)	
Female	55(57.3%)	56(49.6%)	
Acute (vs . Subacute) stage, n (%)	44(45.8%)	67(59.3%)	0.052
Age, years	57.24(17.82)	52.58(18.42)	
Affected leg, n (%)			0.290
Left	67(69.8%)	71(62.8%)	
Right	29(30.2%)	42(37.2%)	
Proximal DVT, n (%)	84(87.5%)	85(75.2%)	0.025
Iliac compression syndrome, n (%)	58(60.4%)	44(38.9%)	0.002
Index DVT + PE (vs. DVT alone) , n (%)	22(22.9%)	20(17.7%)	0.348
Current smoker, n (%)	19(19.8%)	24(21.2%)	0.796
Body mass index, kg/m^2	24.45(3.35)	23.58(3.25)	0.960
Patients with PTS, n	96	113	

Chi-square test or one-way ANOVA was used. Data are mean (SD) or number (%).
PE=Pulmonary Embolism
