# Supplementary material for: Assessment of the Probability of Post-thrombotic Syndrome in Patients with Lower Extremity Deep Venous Thrombosis
Source: Sci Rep. 2018 Aug 23;8:12663. doi: 10.1038/s41598-018-30645-w (PMC6107662; doi:10.1038/s41598-018-30645-w)
Supplement: Supplementary file 2 — Supplement-2 [file 41598_2018_30645_MOESM2_ESM.pdf]

Assessment of the Probability of Post-thrombotic Syndrome in Patients with Lower Extremity Deep Venous Thrombosis

Hao Huang, MD[1],Jian-Ping Gu,MD[1], Hao-Fan Shi,MD[1],Wan-Yin Shi, MD[1], Jing-Yuan Lu, MD[2], Liang Chen, MD[1], Hao-Bo Su, MD[1]

[1]Department of Interventional Radiology, Nanjing First Hospital, Nanjing Medical University, Nanjing, 210001,China

[2]Obstetrics and Gynecology Hospital Affiliated to Nanjing Medical University, Nanjing, 210004, China

Address for Correspondence: Hao-Bo Su, MD, Department of Interventional Radiology, Nanjing First Hospital, Nanjing Medical University, 68 Changle Road , Nanjing, 210001,China

E-mail:nj_dsaalex@126.com

The results of the ROC analysis

(A) Training Cohort

Case Processing Summary

PTS	Valid N (listwise)
Positive[a]	45
Negative	57

Larger values of the test result variable(s) indicate stronger evidence for a positive actual state.

a. The positive actual state is 1.00.

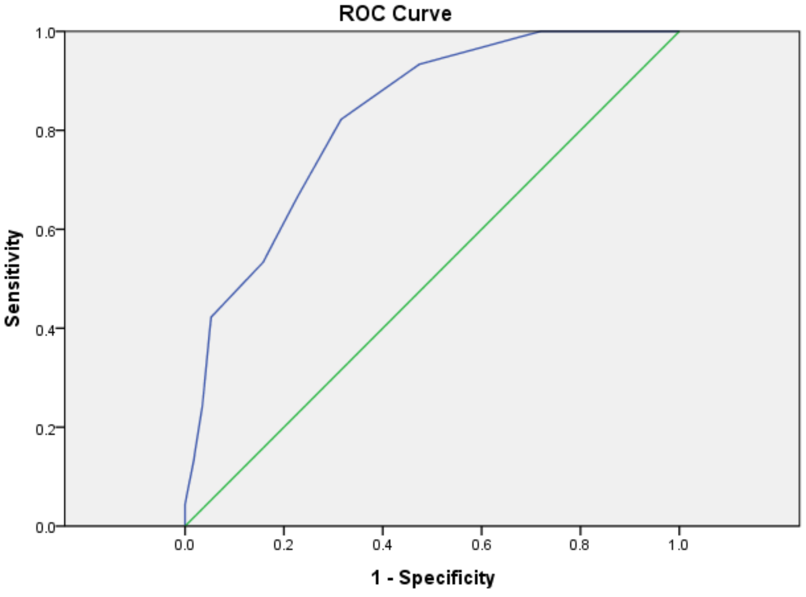

Diagonal segments are produced by ties.

Area Under the Curve

Test Result Variable(s):score

Area	Std. Error[a]	Asymptotic Sig.[b]	Asymptotic 95% Confidence Interval	
			Lower Bound	Upper Bound
.825	.040	.000	.747	.903

The test result variable(s): score has at least one tie between the positive actual state group and the negative actual state group. Statistics may be biased.

a. Under the nonparametric assumption

Area Under the Curve

Test Result Variable(s):score

Area	Std. Error[a]	Asymptotic Sig.[b]	Asymptotic 95% Confidence Interval	
			Lower Bound	Upper Bound
.825	.040	.000	.747	.903

The test result variable(s): score has at least one tie between the positive actual state group and the negative actual state group. Statistics may be biased.

a. Under the nonparametric assumption

b. Null hypothesis: true area = 0.5

Coordinates of the Curve

Test Result Variable(s):score

Positive if Greater Than or Equal To[a]	Sensitivity	1 - Specificity
-7.0000	1.000	1.000
-4.7500	1.000	.737
-3.2500	1.000	.719
-1.7500	.933	.474
-.2500	.822	.316
1.2500	.667	.228
2.7500	.533	.158
4.2500	.422	.053
5.7500	.244	.035
7.2500	.133	.018
10.0000	.044	.000
12.5000	.000	.000

The test result variable(s): score has at least one tie between the positive actual state group and the negative actual state group.

a. The smallest cutoff value is the minimum observed test value minus 1, and the largest cutoff value is the maximum observed test value plus 1. All the other cutoff values are the averages of two consecutive ordered observed test values.

(B)Validation Cohort

Case Processing Summary

PTS	Valid N (listwise)
Positive[a]	45
Negative	57

Larger values of the test result variable(s) indicate stronger evidence for a positive actual state.

a. The positive actual state is 1.00.

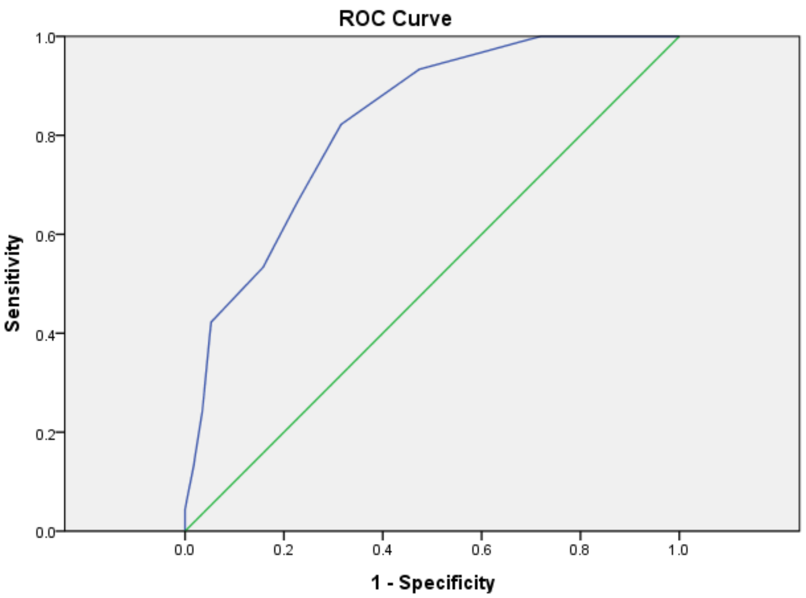

Diagonal segments are produced by ties.

Area Under the Curve

Test Result Variable(s):score

Area	Std. Error[a]	Asymptotic Sig.[b]	Asymptotic 95% Confidence Interval	
			Lower Bound	Upper Bound
.825	.040	.000	.747	.903

The test result variable(s): score has at least one tie between the positive actual state group and the negative actual state group. Statistics may be biased.

a. Under the nonparametric assumption

b. Null hypothesis: true area = 0.5

Coordinates of the Curve

Test Result Variable(s):score

Positive if Greater Than or Equal To[a]	Sensitivity	1 - Specificity
-7.0000	1.000	1.000
-4.7500	1.000	.737
-3.2500	1.000	.719
-1.7500	.933	.474
-.2500	.822	.316
1.2500	.667	.228
2.7500	.533	.158
4.2500	.422	.053
5.7500	.244	.035
7.2500	.133	.018
10.0000	.044	.000
12.5000	.000	.000

The test result variable(s): score has at least one tie between the positive actual state group and the negative actual state group.

a. The smallest cutoff value is the minimum observed test value minus 1, and the largest cutoff value is the maximum observed test value plus 1. All the other cutoff values are the averages of two consecutive ordered observed test values.
